# Comparison of ten predictive equations for estimating lean body mass with dual-energy X-ray absorptiometry in older patients

**DOI:** 10.1259/bjr.20210378

**Published:** 2022-02-11

**Authors:** Tanuj Puri, Glen M Blake

**Affiliations:** School of Biomedical Engineering and Imaging Sciences, King’s College London, St. Thomas’ Hospital, London, United Kingdom; School of Biomedical Engineering and Imaging Sciences, King’s College London, St. Thomas’ Hospital, London, United Kingdom

## Abstract

**Objectives:**

White fat contributes to body weight (BW) but accumulates very little [^18^F]fluorodeoxyglucose ([^18^F]FDG) in the fasting state. As a result, higher standardised uptake values normalised to BW (SUV) are observed in non-fatty tissue in obese patients compared to those in non-obese patients. Therefore, SUV normalised to lean body mass (SUL) that makes tumour uptake values less dependent on patients’ body habitus is considered more appropriate. This study aimed to assess ten mathematical equations to predict lean body mass (LBM) by comparison with dual-energy X-ray absorptiometry (DXA) as the reference method.

**Methods:**

DXA-based LBM was compared with ten equation-based estimates of LBM in terms of the slope, bias and 95% limits of agreement (LOA) of Bland-Altman plots, and Pearson correlation coefficients (r). Data from 747 men and 811 women aged 60–65 years were included.

**Results:**

Gallagher’s equation was optimal in males (slope = 0.13, bias = −2.4 kg, LOA = 12.8 kg and *r* = 0.900) while Janmahasatian’s equation was optimal in females (slope = 0.14, bias = −0.9 kg, LOA = 10.7 kg and *r* = 0.876). Janmahasatian’s equation performed slightly better than Gallagher’s in the pooled male and female data (slope = 0.00, bias = −1.6 kg, LOA = 12.3 kg and *r* = 0.959).

**Conclusions:**

The Gallagher and Janmahasatian equations were optimal and almost indistinguishable in predicting LBM in subjects aged 60–65 years.

**Advances in knowledge:**

Determination of the optimum equation for predicting lean body mass to improve the calculation of SUL for [^18^F]FDG PET quantification.

## Introduction

[^18^F]fluorodeoxyglucose ([^18^F]FDG) positron emission tomography (PET) scans are commonly used for imaging cancer patients.^1^ PET images are interpreted quantitatively using standardised uptake values (SUV).^2^ SUV provides a measure of tracer uptake per unit volume within a region of interest (ROI) over a small interval at a particular time t, calculated as follows:

$$SUV=\frac{Tracer\;Uptake\;Within\;ROI\left(units:kBq/ml\right)*Body\;Weight\left(units:g\right)}{Injected\;Activity\left(units:kBq\right)}$$

It is used to express the level of tracer uptake in tumours for various clinical and research purposes.^3^ Assumptions associated with SUV, their advantages and disadvantages have been discussed previously.^4–6^

Tumour activity normalisation using lean body mass (LBM) or body surface area (BSA) has been suggested to be better than using body weight (BW) in various [^18^F]FDG PET oncology studies.^7–13^ The main rationale for using LBM for normalising tumour activity is that very low uptake of [^18^F]FDG is observed in white fat in the fasting state.^7,8^ The observation that fat contributes to BW but accumulates very little [^18^F]FDG in the fasting state has been considered to be a reason for higher SUV in non-fatty tissue in obese patients compared to non-obese patients. Therefore, the use of a normalisation factor such as LBM or BSA may be more appropriate.

Many different methods exist to measure LBM in humans including whole-body dual-energy X-ray absorptiometry (DXA), air displacement plethysmography, skinfold thickness and bioimpedance.^14^ Measuring LBM using imaging in clinical trials is challenging in terms of standardisation, cost, scan time and radiation dose to patients. Therefore, the use of mathematical equations to predict LBM based on patients’ height, weight, age, ethnicity and sex is a practical and cost-effective alternative. For this reason, predictive equations for assessing LBM have long been a topic of interest to researchers.^15,16^

Many different equations to predict LBM have been used in the PET literature. However, a thorough investigation of predictive equations to assess LBM in a large British population in greater detail is missing. Therefore, this study aimed to assess ten predictive equations for calculating LBM, some of which are commonly used in the PET literature, to find the most accurate equation by comparison with DXA as the reference method.

## Methods and materials

Whole-body DXA scans are two-dimensional projection scans in which X-ray attenuation through the body is measured at two different photon energies enabling a pixel-by-pixel estimate of the areal densities (units g/cm^2^) of two different types of tissue.^17^ After first flagging those pixels that include bone, the remaining pixels are used to measure the areal densities of lean and fat tissue. In bone pixels, bone mineral density and the areal densities of lean and fat are measured on the assumption that the percentage body fat (%BF) can be interpolated from the adjacent non-bone pixels. DXA manufacturers fine-tune their algorithms for deriving lean and fat mass by calibrating them against a suite of more sophisticated methods.^14,17^ Although DXA is not a gold standard method for measuring LBM, the low radiation dose, low cost and widespread availability make it an attractive method for many studies.

### Data demographics

Whole-body DXA measurements of LBM in 1,558 subjects (747 men, 811 women) acquired with Hologic Discovery densitometers (Hologic Inc., Malborough, MA) were obtained from the Medical Research Council (MRC) National Survey of Health and Development (NSHD).^18^ All were acquired in the period 2006–2011 using the same DXA scan mode. Demographic details of this cohort are described in Table 1. These data were retrospectively analysed to compare equation-based predicted LBM and DXA-based LBM to find the optimum predictive equation. Because the brain is enclosed by bone, there are no appropriate non-bone pixels for inferring its composition and the manufacturer’s software makes assumptions about its fat content. For this reason, the results of DXA scans are sometimes reported excluding the head. However, since [^18^F]FDG is taken up by the brain, for the present study we choose to compare the predictive equations with DXA data that included the head.

**Table 1.** Demographic details of the MRC National Survey of Health and Development DXA body composition study cohort^17^ expressed as mean and range

Sex	N	Mean Age (range) in yrs	Mean Weight (range) in kg	Mean Height (range) in cm
Males	747	63 (60–65)	85.3 (50.6–128.5)	175.3 (156.7–195.7)
Females	811	63 (60–65)	72.3 (38–136.5)	162.2 (144.3–179.0)
Both sexes	1558	63 (60–65)	78.5 (38–136.5)	168.5 (144.3–195.7)

DXA, dual energy X-ray absorptiometry; N, number of subjects; cm, centimeters; kg, kilograms; yrs, years.

### Predictive equations

The equations examined were those of Hume,^19^ Hume & Weyers,^20^ James & Waterlow,^21^ Hallynck et al.,^22^ Boer,^15^ Deurenberg et al.,^23^ Zasadny & Wahl,^7^ Morgan & Bray,^16^ Gallagher et al.^24^ and Janmahasatian et al.^25^ A brief description of each equation is provided in Table 2 and a detailed description is given in the Supplementary Material 1. These ten equations were selected as ones commonly found in the PET literature for normalising SUV to LBM.

**Table 2.** Demographic details of the ten study cohorts used to develop the predictive equations expressed as mean and range

Eq. No.	Ref	Sex	N	Mean Age (range) - y	Mean Weight (range) - kg	Mean Height (range) - cm	Predictive Equations
Eq1	^19^	M	29	61.0 (40–77)	75.7 (43–133)	169.6 (151–188)	LBM = 0.32810*W(kg)+0.33929*H(cm) – 29.53
		W	27	60.0 (37–80)	68.4 (45–116)	156.3 (139–182)	LBM = 0.29569*W(kg)+0.41813*H(cm) – 43.29
Eq2	^20^	M	30	54.5 (35–71)	71.8 (36–122)	170.2 (132–185)	LBM= [0.296785*W(kg)+0.194786*H(cm) – 14.01]*100/73
		W	30	53.7 (33–84)	64.7 (32–108)	156.3 (144–170)	LBM = [0.183809*W(kg)+0.344547*H(cm) – 35.27]*100/73
Eq3	^21^	M	-	-	-	-	%BF = 1.281*[W(kg)/H(m)^2] – 10.13
		W					%BF = 1.48*[W(kg)/H(m)^2] – 7.0
Eq4	^22^	M	-	-	-	-	LBM = 1.10*W(kg) – 128*[W(kg)/H(cm)]^2
		W					LBM = 1.07*W(kg) – 148*[W(kg)/H(cm)]^2
Eq5	^15^	M	47	Not provided (21–72)	-	-	LBM = 0.407*W(kg)+26.7*H(m) – 19.2
		W	40	Not provided (19–68)	-	-	LBM = 0.252*W(kg)+47.3*H(m) – 48.3
Eq6	^23^	M	521	-	-	-	%BF = 1.2*BMI + 0.23*AGE(yrs) – 16.2
		W	708				%BF = 1.2*BMI + 0.23*AGE(yrs) – 5.4
Eq7	^7^	M	-	-	-	-	LBM = 48+1.06*[H(cm) – 152]
		W	28	54 (29–75)	72 (45–107)		LBM = 45.5+0.91*[H(cm) – 152]
Eq8	^16^	M	-	-	-	-	LBM = 1.10*W(kg) – 120*[W(kg/H(cm)]^2
		W					LBM = 1.07*W(kg) – 148*[W(kg)/H(cm)]^2
Eq9	^24^	M	291	49.2 (Not provided)	80.2 (Not provided)	176 (Not provided)	%BF = 48.1–848/BMI + 0.084*AGE(yrs)+39/BMI
		W	380	51.8 (Not provided)	66.3 (Not provided)	163 (Not provided)	%BF = 64.5–848/BMI + 0.079*AGE(yrs)
Eq10	^25^	M	168	42.1 (18–82)	106.0 (60–217)	177.4 (159–208)	FFM = 9270*W(kg)/(6680 + 216*BMI)
		W	205	41.2 (19–79)	88.3 (41–196)	164.2 (137–187)	FFM = 9270*W(kg)/(8780 + 244*BMI)

AGE(yrs), Age in years; %BF, Percentage body fat; BMI, Body mass index; Eq, Equation; H(cm), height in cemtimeters; H(m), height in meters; LBM, Lean body masss; *M*, males; *N*, number of subjects; Ref, reference number in text; *W*, females; W(kg), weight in kilograms; cm, centimeters; kg, kilograms; yrs, years.

### Statistical analysis

R software^26^ was used to analyse combined data of males and females, as well as males and females separately. To assess the appropriateness of parametric statistical tests, DXA-based LBM, the results of the predictive equations and their differences were tested for normality using the Shapiro-Wilk test. The differences between the DXA-based LBM and equation-based LBM were then assessed in terms of four parameters: (i) the slope, (ii) bias and (iii) 95% limits of agreement (LOA) of their respective Bland-Altman (BA) plots,^27^ and (iv) the Pearson correlation coefficient.

We first chose the optimum equation in terms of each of these four parameters and eliminated other equations which had values outside the 95% confidence intervals (95% CI). The remaining equations were considered statistically indistinguishable from each other. (i) Based on the slope of the regression line fitted to the BA plots, equations were eliminated if the 95% CI of the slope did not include zero. (ii) Based on the bias, equations were eliminated if the 95% CI of the bias did not include zero. (iii) Based on the LOA, the standard deviation (SD) of the Y-axis values in the BA plot was obtained by dividing LOA by 3.92 (*i.e.,* 2 × 1.96). The SD of each equation was then compared with the lowest value using the F-test and equations were excluded for those with *p* < 0.05. (iv) Based on the Pearson correlation coefficient, the equation with the highest value was taken and compared with 95% CI of the r-values for the other equations to check if they included or excluded this highest value.

## Results

The results of slope, bias and LOA of the differences of the BA plots and correlation coefficients for all equations for males and females and their pooled data are summarised in Table 3. For the pooled data, in terms of slope, Eqs 7, 8, 9 and 10 all showed values with 95% CI consistent with zero. For bias, Eqs 1, 6 and 10 were all consistent with zero. For LOA, Eq 9 was best with an SD of 2.93 kg. The next best was Eq 10 (SD: 3.08 kg, *p* = 0.029). For the correlation coefficient, Eq 9 gave the largest value (*r* = 0.962), but Eqs 2, 5, 6 and 10 were within the 95% CI. When males and females were analysed separately, Eq 9 was optimal in males and Eq 10 in females (Table 3).

**Table 3.** The slope, bias and limit of agreement (LOA) obtained from the Bland-Altman plots of the differences between the LBM from DXA and each of the predictive equations. Pearson correlations are also reported between LBM from DXA and each of the predictive equations. Results are shown for pooled males and females (*M* + W), males only (M) and females only (W). *highlights the optimum fit. +indicates other fits that are statistically consistent with the optimum

	*M* + W	*M* + W	*M* + W	*M* + W	M	M	M	M	W	W	W	W
	Slope	Bias	LOA	Pearson	Slope	Bias	LOA	Pearson	Slope	Bias	LOA	Pearson
Eq1: Hume^19^	0.32	−1.1*	16.9	0.945	0.32	1.8*	14.2+	0.890+	0.08	−3.8	11.3	0.861+
Eq2: Hume & Weyers^20^	0.13	−3.5	13.0	0.957+	0.20	−2.5+	13.0+	0.899+	0.15	−4.3	12.1	0.840
Eq3: James & Waterlow^21^	0.15	−4.1	14.2	0.948	0.21	−3.3	13.6+	0.889+	0.33	−4.9	14.0	0.776
Eq4: Hallynck ^22^	0.15	−4.1	14.2	0.948	0.21	−3.2	13.6+	0.882	0.33	−4.9	14.0	0.776
Eq5: Boer^15^	0.14	−3.6	13.1	0.957+	0.20	−2.6+	13.0+	0.899+	0.15	−4.5	12.1	0.840
Eq6: Deurenberg^23^	0.14	1.5+	13.9	0.950+	0.31	2.0+	14.2	0.887+	0.42	1.0+	13.3	0.810
Eq7: Zasadny & Wahl^7^	0.02+	−12.8	25.0	0.830	0.12	−12.9	27.5	0.525	0.10	−12.7	22.4	0.451
Eq8: Morgan & Bray^16^	0.05+	−5.0	13.6	0.949	0.13	−5.1	13.1+	0.896+	0.33	−4.9	14.0	0.776
Eq9: Gallagher^24^	0.04+	−2.4	11.8*	0.962*	0.13	−2.4+	12.8*	0.900*	0.17	−2.3	10.7*	0.877*
Eq10: Janmahasatian^25^	0.00*	−1.6+	12.3	0.959+	0.21	−2.3+	13.3+	0.895+	0.14	−0.9*	10.7*	0.876+

DXA, Dual-energy X-ray ansorptiometry; Eq, Equation; LBM, Lean body mass; LOA, Limit of agreement.

**Figure 1.**
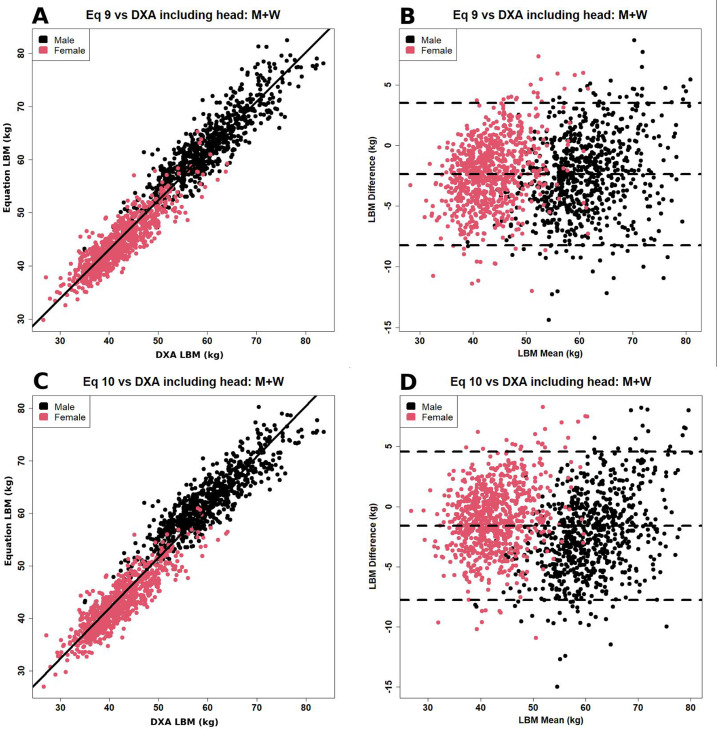
(**A**) Scatter and (**B**) Bland-Altman plot of the relationship between lean body mass measured by dual-energy X-ray absorptiometry (DXA) and predicted by the Gallagher equation (equation 9 in Table 2).^24^ (**C**) and (**D**): Similar graphs for the Janmahasatian equation (equation 10 in Table 2).^25^

Overall, for the male, female and pooled data Eqs 9 and 10 were optimal and were not statistically distinguishable. Figure 1 shows the scatter and Bland-Altman plots for these two equations. Similar plots for the other eight equations are shown in Figures S1 to S4 in the Supplementary Material 1.

## Discussion

In this study, we have used DXA data to compare ten mathematical equations that predict LBM and to choose an optimal equation for use in PET oncology studies.

It is known that body composition varies with race, gender, age and body size. In adults, weight gain mostly represents the accumulation of body fat. For this reason, many researchers have aimed to develop an age-specific formula to predict %BF. However, Eqs 1-5, 7, 8 and 10 in Table 2 do not include age as an independent variable, and these equations may be too simple to model LBM over a wide age range, which might explain their poorer predictions. On the other hand, Eq 9 provided better predictions as it included age, sex and body mass index (BMI) as the independent predictors of LBM. Interestingly, Eq 10 agreed well with DXA although it does not include age as an independent predictor of LBM despite including data from subjects with a wide age range of 18–82 years for modelling the equation. Although our study shows that Eq 10 works well in the 60–65 year age group, we cannot comment on how well it works for other age ranges.

There are technical reasons why some of the equations under consideration might perform poorly. Hume & Weyers^20^ modelled Eq 2 on the assumption that total body water is 73% of fat-free mass (FFM). Nyman^28^ noticed that Eq 3 was not appropriate for predicting LBM in obese population as the model plateaued at a BMI of 37 kg/m^2^ in females and 43 kg/m^2^ in males before decreasing at higher BMI. Eqs 1-5 and Eqs 7-8 were regressed based on small datasets which may contribute to some degree of error in these models. In contrast, Eq 6 is based on 1,229 subjects, Eq 9 on 671 subjects, and Eq 10 on 373 subjects, which may have contributed to making them more reliable than others. The better agreement of Eq 9 and 10 may also be because these two equations were partly derived using DXA measurements while others were not.

Carnevale et al.^29^ compared Eq 1 in 100 subjects against the DXA-based LBM. Their results (*r* = 0.83, BA plot bias: 1.36 kg, LOA: 14.78 kg) were similar to ours (*r* = 0.95, BA plot bias: −1.10 kg, LOA: 16.89 kg). More importantly, a large slope was visible in the BA plots between Eq 1 and DXA values in their study, consistent with our results (slope = 0.32). This was the primary reason why we dropped Eq 1 as a method for predicting LBM in this analysis. Another study analysed data for 1,655 older males over the age of 65 and reported Eq 10 to be optimal when compared with DXA data.^30^ However, these authors did not consider Eq 9.

Many studies have suggested that measurement of SUV normalised to lean body mass (SUL) may have advantages over the conventional normalisation to BW for [^18^F]FDG PET imaging of solid tumours and lymphoma.^31^ ^32^ Hence, there is interest in equations using factors such as BW, height, age and sex to predict LBM. It is important to note that cancer applications using PET tend to include older patients. That was the reason why we evaluated the predictive equations with a population of age between 60 and 65 years, which is representative of the ages of patients at our centre.

Other factors also require consideration. The fact that [^18^F]FDG does not significantly accumulate in white fat in the fasting state suggests that changes in fat and muscle composition in the early stages of cancer and during chemotherapy may be relevant. Cachexia has been reported in patients with breast, colorectal, urological and pancreatic cancers, lymphoma and pediatric patients. Therefore, the use of DXA or air displacement plethysmography may be more accurate in assessing body composition than predictive equations in these patients.

Limitations of the study include: (1) we assessed only a small number of equations to predict LBM as it was not practical to include all equations in the literature; (2) Our analysis presumes that DXA total body scanning is a suitable reference method for measuring LBM; (3) Most of the equations were developed for white populations and may not be appropriate for other ethic groups in the United Kingdom; (4) This study examines the differences between predictive equations for LBM but not their application to the correction of clinical PET measurements where other factors such as calibration, injected activity and uptake time^33^ may be important.

## Conclusion

In conclusion, the Gallagher and Janmahasatian equations performed optimally and were almost indistinguishable when compared to the DXA-based LBM in subjects with ages between 60 and 65 years. Application of these equations to a large [^18^F]FDG dataset is required to test the hypothesis that normalisation of tracer uptake to LBM may perform better for PET quantification than normalisation to body weight.

## Supplementary Material

bjr.20210378.pdf
